# The Cholinergic and Adrenergic Autocrine Signaling Pathway Mediates Immunomodulation in Oyster *Crassostrea gigas*

**DOI:** 10.3389/fimmu.2018.00284

**Published:** 2018-02-26

**Authors:** Zhaoqun Liu, Lingling Wang, Zhao Lv, Zhi Zhou, Weilin Wang, Meijia Li, Qilin Yi, Limei Qiu, Linsheng Song

**Affiliations:** ^1^Liaoning Key Laboratory of Marine Animal Immunology, Dalian Ocean University, Dalian, China; ^2^Laboratory of Marine Fisheries Science and Food Production Processes, Qingdao National Laboratory for Marine Science and Technology, Qingdao, China; ^3^Key Laboratory of Experimental Marine Biology, Institute of Oceanology, Chinese Academy of Sciences, Qingdao, China; ^4^University of Chinese Academy of Sciences, Beijing, China; ^5^Key Laboratory of Tropical Biological Resources of Ministry of Education, Hainan University, Haikou, China

**Keywords:** *Crassostrea gigas*, autocrine/paracrine, hemocyte, neurotransmitter, membrane receptor, immune regulation

## Abstract

It is becoming increasingly clear that neurotransmitters impose direct influence on regulation of the immune process. Recently, a simple but sophisticated neuroendocrine–immune (NEI) system was identified in oyster, which modulated neural immune response *via* a “nervous-hemocyte”-mediated neuroendocrine immunomodulatory axis (NIA)-like pathway. In the present study, the *de novo* synthesis of neurotransmitters and their immunomodulation in the hemocytes of oyster *Crassostrea gigas* were investigated to understand the autocrine/paracrine pathway independent of the nervous system. After hemocytes were exposed to lipopolysaccharide (LPS) stimulation, acetylcholine (ACh), and norepinephrine (NE) in the cell supernatants, both increased to a significantly higher level (2.71- and 2.40-fold, *p* < 0.05) comparing with that in the control group. The mRNA expression levels and protein activities of choline O-acetyltransferase and dopamine β-hydroxylase in hemocytes which were involved in the synthesis of ACh and NE were significantly elevated at 1 h after LPS stimulation, while the activities of acetylcholinesterase and monoamine oxidase, two enzymes essential in the metabolic inactivation of ACh and NE, were inhibited. These results demonstrated the existence of the sophisticated intracellular machinery for the generation, release and inactivation of ACh and NE in oyster hemocytes. Moreover, the hemocyte-derived neurotransmitters could in turn regulate the mRNA expressions of tumor necrosis factor (TNF) genes, the activities of superoxide dismutase, catalase and lysosome, and hemocyte phagocytosis. The phagocytic activities of hemocytes, the mRNA expressions of TNF and the activities of key immune-related enzymes were significantly changed after the block of ACh and NE receptors with different kinds of antagonists, suggesting that autocrine/paracrine self-regulation was mediated by transmembrane receptors on hemocyte. The present study proved that oyster hemocyte could *de novo* synthesize and release cholinergic and adrenergic neurotransmitters, and the hemocyte-derived ACh/NE could then execute a negative regulation on hemocyte phagocytosis and synthesis of immune effectors with similar autocrine/paracrine signaling pathway identified in vertebrate macrophages. Findings in the present study demonstrated that the immune and neuroendocrine system evolved from a common origin and enriched our knowledge on the evolution of NEI system.

## Introduction

The nervous and endocrine systems regulate the immune system through releasing neurotransmitters, neuropeptides, and endocrine hormones as they modulate the other physiological activities (1). Recently, it has been realized that neurotransmitters derived from sources outside the nervous and endocrine systems, especially from immune system, can also serve as immunomodulators (2). The immune cell-derived neurotransmitters can bind to autocrine receptors on their own, exerting a considerable and reciprocal influence on the function of immune system (3). By far, such neuroendocrine autocrine/paracrine signaling has been well studied in vertebrates. The dendritic cells, leukocytes, and lymphocytes can synthesize and/or release classical neurotransmitters, including acetylcholine (ACh), dopamine (DA), serotonin (5-HT), and glutamate (4–7). These neurotransmitters in turn exert diverse effects during inflammation *via* autocrine/paracrine signaling pathways (2). For instance, DA and glutamate are able to interact directly with T-cell expressed receptors, leading to the activation or suppression of various T-cell functions including cytokine secretion, proliferation, integrin-mediated adhesion, and migration (8–12).

Comparing with model species, study on the neuroendocrine immune (NEI) regulation in invertebrates is still at the very outset. Most of the previous research focused on the immunomodulation of neurotransmitters released from neuroendocrine system. For instance, catecholamines (CAs), ACh, 5-HT, γ-aminobutyric acid (GABA), histamine, enkephaline (ENK), glutamic acid (GA), neuropeptide Y (NPY), and nitric oxide (NO) have been identified from the nervous and endocrine tissues in mollusks (13, 14). These neurotransmitters conduct neural immune regulation through a nervous-hemocyte neuroendocrine immunomodulatory axis (NIA)-like pathway, modulating both cellular and humoral immune activities in mollusk (15). Astonishingly, recent studies have illustrated that molluskan immune system can also synthesize neurotransmitters and may conduct autocrine/paracrine immune regulation. The key enzymes for CAs synthesis [dopamine β-hydroxylase (DBH)] and ACh degradation [acetylcholinesterase (AChE)] are reported to be present in molluskan hemocytes (16–18), and ACh and NE can be detected in hemolymph (17, 19, 20). These findings imply the existence of cholinergic and adrenergic autocrine/paracrine pathways in molluskan hemocytes, which mediate neural immunomodulation at cellular level.

Circulating immunocytes play the most important roles in both neural immune regulation generated by nervous-derived neurotransmitters and autocrine/paracrine immune regulation conducted by cell-derived neurotransmitters (2, 21). Moreover, in both vertebrates and invertebrates, immunocytes act as fundamental players in the crosstalk between the NEI systems since they display significant overlap in molecular components and physiological functions (22). Numerous morphological and functional studies have indicated that there is a common pool of molecules shared by the immune and neuroendocrine systems, and there should be a common evolutionary origin for the two systems in both invertebrates and vertebrates (23). Therefore, the neuroendocrine autocrine/paracrine signaling existing in immunocytes exhibits perfect example for this hypothesis, and study on the neuroendocrine autocrine/paracrine pathway in invertebrate hemocytes can also provide insights into the evolution of the NEI system.

The complexity of immune and neuroendocrine components in mammals has prompted corresponding studies in simpler models. Among these, mollusks have been considered as valuable model for analyzing the basic patterns of the immune and neuroendocrine interactions (22) since they are the most primitive organisms evolved with a sophisticated NEI system (24), in which the circulating hemocytes are described as the “immune-mobile brain” for their ability to recognize a variety of stimuli and to set up sophisticated responses (25). Most of the previous studies focused mainly on the immune response of hemocytes mediated by neurotransmitters, while their potentials to produce neurotransmitters were rarely mentioned. The aims of the present study are to (1) confirm the *de novo* synthesis of cholinergic and adrenergic neurotransmitters in oyster hemocytes, (2) investigate the immunomodulation of cholinergic and adrenergic systems mediated by hemocytes, and (3) evaluate the immunological activity of autocrine/paracrine neurotransmitters at cellular level. Investigation on the cholinergic and adrenergic autocrine immunomodulation in oyster hemocytes will enrich our knowledge about the common origin of NEI systems, as well as the evolution of NEI system.

## Materials and Methods

### Oysters and Primary Culture of Hemocytes

Oysters *Crassostrea gigas* (with an average of 150 mm in shell height) were collected from a local farm in Qingdao, Shandong Province, China, and maintained in aerated seawater at 18°C for 2 weeks before processing.

Oyster hemolymph was aspirated from the blood sinus with a thin syringe, and centrifuged at 800 *g* to harvest the hemocytes. The hemolymph from five oysters was pooled together, and there were three parallel pools for each test. Primary culture of oyster hemocytes was carried out based on the protocol described by Jiang et al. (26). The cell viability was detected by Trypan Blue exclusion technique using commercial kit (Beyotime Biotechnology).

All animal-involving experiments of this study were approved by the Ethics Committee of the Institute of Oceanology, Chinese Academy of Sciences.

### Lipopolysaccharide (LPS) Stimulation, Antagonist Treatment, and Sample Collection

For the LPS stimulation experiment, hemocytes were incubated with 100 ng mL^−1^ of LPS origin from *Escherichia coli* 0111:B4 (Sigma) for 30 min and 1 h, respectively, while the same volume of PBS (Gibco, pH 7.4) was added in the negative control (Neg-Ctrl) group.

The incubation of antagonists for neurotransmitter receptors on oyster hemocytes was performed according to the description in previous studies to explore whether hemocyte-derived neurotransmitters could exert autocrine/paracrine immune regulation *via* cell-surface receptors (27–29). In the present study, non-selective nicotinic acetylcholine receptor (nAChR) antagonist mecamylamine hydrochloride (Tocris Bioscience) was employed in the non-selective nAChR antagonists group, while α-7 nAChR antagonist α-Bungarotoxin (Tocris Bioscience) was added in the α-7 nAChR antagonists group. In order to block the muscarinic acetylcholine receptors (mAChRs) on the hemocyte surface, five antagonists including pirenzepine (Tocris Bioscience), AFDX 116 (Tocris Bioscience), 4-DAMP (Tocris Bioscience), PD102807 (Tocris Bioscience), and darifenacin (Sigma) specific for the m1 to m5 mAChRs, respectively, were used in the m1–m5 mAChRs antagonists groups (30). In addition, the mixture of doxazosin mesylate [specific antagonists for α-1 adrenergic receptor (A1AR), Tocris Bioscience] and idazoxan yohimbine [specific antagonists for α-2 adrenergic receptor (A2AR), Tocris Bioscience] were added in the α-antagonists group to inhibit the binding activities of α adrenergic receptors (AARs), while propranolol (Tocris Bioscience), specific antagonist for β-adrenergic receptor (BAR), was added in the β-antagonist group to block BARs (31–33). Furthermore, in order to explore the synergistic immunomodulation of ACh and NE, the mixture of different kinds of antagonists was employed to block all AChRs and ARs. In the α-7 + m1-5 group, α-7 nAChR antagonist (α-Bungarotoxin) and m1 to m5 mAChR antagonists (pirenzepine, AFDX 116, 4-DAMP, PD102807, and darifenacin) were added, while in α + β group, A1AR antagonists (doxazosin mesylate), A2AR antagonist (idazoxan yohimbine) and BAR antagonist (propranolol) were used to block the ARs and BRs on hemocyte surface. All antagonists were employed at a final concentration of 10.0 µmol L^−1^ and incubated with hemocytes for 1 h before LPS stimulation (100 ng mL^−1^). Hemocytes incubated with Leibovitz-15 (L-15) medium for 1 h and subsequently with LPS stimulation were employed as Vehicle group, and cells incubated with PBS instead of antagonists or LPS were treated as the Neg-Ctrl group.

Hemocytes from different groups were collected at 3 h after LPS stimulation for the subsequent determinations of phagocytosis, mRNA expressions and enzyme activities. Three replicates were considered for each assay.

### RNA Extraction and Quantitative Real-time PCR

Trizol reagent was used to extract the total RNA from oyster hemocytes. DNase I (Promega) and oligo (dT)-adaptor were then employed to synthesize the cDNA library. Next, the constructed cDNA library was used to evaluate the mRNA expression levels of three oyster tumor necrosis factors (TNFs) including CGI_10005109, CGI_10005110, and CGI_10006440, by using SYBR green quantitative real-time PCR technique (34). The amplified fragment (168 bp) of oyster elongation factor (CgEF, CGI_10012474) was employed as the endogenous control. The primers used in the present study (Table 1) have been verified in previous research according to the dilution curve detected by the 7500 real-time PCR system (Applied Biosystem) (35). Three replicates were detected for each sample and all data were shown in terms of relative expression using the 2^−ΔΔCt^ method (36).

**Table 1:** Sequences of the primers used in the experiment.

Primer	Sequence (5′-3′)	Sequence information
P1 (forward)	CGCAATGGTCGCTTGGTGGTC	Real-time CgTNF (CGI_10005109) primer
P2 (reverse)	CGTAGGGGCGGAAGGTCTCG	Real-time CgTNF (CGI_10005109) primer
P3 (forward)	CAACGGTCTAACTTACCATCCAAAC	Real-time CgTNF (CGI_10005110) primer
P4 (reverse)	TGGTGGTAGATAAAATGGGACAGTG	Real-time CgTNF (CGI_10005110) primer
P5 (forward)	ATTGGAGCACCTGGAGGATAAG	Real-time CgTNF (CGI_10006440) primer
P6 (reverse)	CAGTCTTCCGTGCTGGTATTTC	Real-time CgTNF (CGI_10006440) primer
P7 (forward)	TGAGTCCAGATTCCTTTATCCAGTTAG	Real-time CgChAT (CGI_10023267) primer
P8 (reverse)	TCCAAAGCATCTGGGGTGTTAG	Real-time CgChAT (CGI_10023267) primer
P9 (forward)	GGTAATAACGAAAGGAAACGAAG	Real-time CgDBH (CGI_10027734) primer
P10 (reverse)	CACCGATAACTTCCCGACAC	Real-time CgDBH (CGI_10027734) primer
P11 (forward)	ACCTATTCAATCATCGCTCCTCC	Real-time CgAChE (CGI_10019411) primer
P12 (reverse)	TCTCTTTATACGTGTGAAGGGGC	Real-time CgAChE (CGI_10019411) primer
P13 (forward)	AGACAACTGATGGAGTGACGGTG	Real-time CgMAO (CGI_10022845) primer
P14 (reverse)	TCCAAAAAGGGGTCTTGTAGTAGC	Real-time CgMAO (CGI_10022845) primer
P15 (forward)	ATCCTTCCTCCATCTCGTCCT	Real-time CgEF (CGI_10012474) primer
P16 (reverse)	GGCACAGTTCCAATACCTCCA	Real-time CgEF (CGI_10012474) primer
M13-47	CGCCAGGGTTTTCCCAGTCACGAC	pMD18-T simple vector primer
RV-M	GAGCGGATAACAATTTCACACAGG	pMD18-T simple vector primer
P17 (forward)	GGCCACGCGTCGACTAGTACT_17_	Oligo(dT)-adaptor

### Quantification of ACh and NE in Hemocyte Supernatants

The contents of ACh and NE in the primarily cultured hemocyte supernatants after LPS stimulation were determined using the ACh and NE ELISA kit (Abnova) according to previous reports (26, 37). The quantification of samples was conducted by comparing the absorbance with a reference curve. There were about 10^5^ cells in each well, and three replicates were employed for each control and experimental group.

### Measurements of Enzyme Activity in Oyster Hemocytes

The activities of two key ACh/NE synthesis enzymes in oysters, choline O-acetyltransferase (CgChAT) and dopamine β-hydroxylase (CgDBH), as well as two key ACh/NE degradation enzymes, acetylcholinesterase (CgAChE) and monoamine oxidase (CgMAO), were measured in the present study. CgChAT activity was determined using a kit (Jiancheng, A079; Nanjing) according to its protocol. The cell lysates was first incubated with reagents 1–6 provided by the kit at 37°C for 5 min, followed by terminating the reaction in boiling water for 2 min. The evaluation of CgDBH activity was conducted with two enzymatic reactions according to the previous description (38). In the first reaction, tyramine was added to the homogenate, and it could be converted to octopamine by DBH. In the subsequent second reaction, the enzymatically formed octopamine was further converted to N-methyl octopamine by the added PNMT. S-adenosylmethionine served as a methyl donor, and the amount of C^14^-N-methyl octopamine was proportional to DBH activity. In addition, CgAChE activity was determined based on the colorimetric method (39). First, 330 µL of PBS, 20 µL of 5,5′-dithiobis-(2-nitrobenzoic acid) (DTNB, 0.0076 mol L^−1^) working as chromogenic agent, as well as 100 µL of hemocyte lysates were mixed and placed in a 96-well plate. Then, 10 µL of acetylthiocholine iodide (ATC, 0.076 mol L^−1^) were added and the enzyme activity was evaluated. Wells without ATC or cell lysates were designated as controls and spontaneous substrate hydrolyzates was determined. The absorbance of 2-nitro-5-thiobenzoate anion was measured at 405 nm. Finally, the activity of CgMAO was determined as described by Zhou et al. (16), taking the metabolism rate of serotonin as its activity. Three replicates were conducted for each assay.

The activities of superoxide dismutase (SOD), catalase (CAT), and lysozyme (LYZ) in hemocyte lysates were then measured. And the determinations were conducted using kits from Jiancheng (Nanjing).

### Determination of Hemocyte Phagocytic Activity

The phagocytic activity of hemocytes was measured according to the previous report (34). The concentration of hemocytes, resuspended in seawater, was adjusted to 1.0 × 10^6^ cells mL^−1^ and 500 µL of hemocyte resuspension was incubated with 5 µL of dead, FITC-labeled *Vibrio splendidus* (1.0 × 10^9^ CFU mL^−1^) at room temperature for 1 h. After washing in PBS, hemocytes were centrifuged to discard un-ingested bacteria. The intensity of FITC fluorescence was measured by flow cytometry, and the phagocytic activity was calculated as (number of phagocytic cells with ingested bacteria)/(number of phagocytes). Three replicates were determined for each assay.

### Statistical Analysis

All data were presented as means ± SD, and subjected to one-way analysis of variance in SPSS software, followed by multiple comparisons (S-N-K). Differences were considered significant at *p* < 0.05.

## Results

### Concentration Changes of ACh and NE in Hemocyte Supernatants after LPS Stimulation

**Figure 1:**
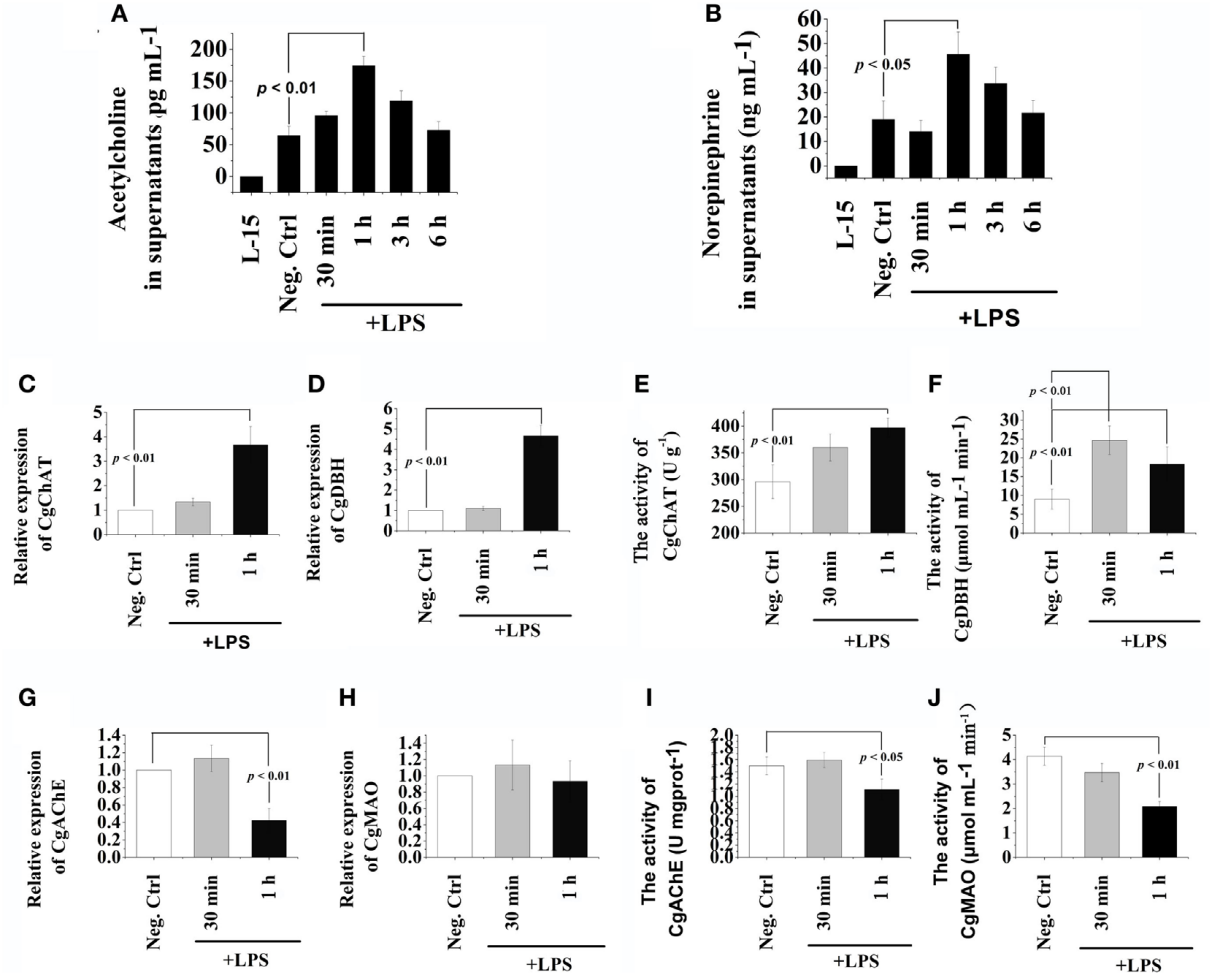
Release of acetylcholine (Ach) and norepinephrine (NE) from oyster hemocytes and the presence of ACh/NE-producing enzymes in hemocytes. **(A,B)** After isolation and primary cell culture, hemocytes were incubated with 100 ng mL^−1^ of lipopolysaccharide (LPS) *in vitro*, while the same volume of PBS was added in the negative control (Neg-Ctrl) group. Hemocytes incubated with Leibovitz-15 (L-15) medium for 1 h and subsequently with LPS stimulation were employed as Vehicle group. Cellular supernatant fluids were collected as a function of time thereafter and then analyzed by enzyme-linked immunosorbent assay for acetylcholine **(A)** and norepinephrine **(B)**. **(C–H)** After stimulation with 100 ng mL^−1^ of LPS *in vitro*, mRNA from hemocytes were sampled and subjected to real-time PCR analysates for choline O-acetyltransferase **(C)**, dopamine beta-hydroxylase **(D)**, acetylcholinesterase **(G)**, and monoamine oxidase **(H)**. **(E,F,I,J)**, After stimulation with 100 ng mL^−1^ of LPS *in vitro*, protein from hemocytes were extracted and subjected to enzyme activity measurement of choline O-acetyltransferase **(E)**, dopamine beta-hydroxylase **(F)**, acetylcholinesterase **(I)**, and monoamine oxidase **(J)**. Each bar represents *N* = 6 samples. All data are presented as means ± SD.

The concentration of ACh and NE in supernatants of hemocyte at 30 min, 1, 3, and 6 h after LPS stimulation was quantified to evaluate the *de novo* production of cholinergic and adrenergic neurotransmitters (Figure 1). The concentrations of ACh and NE in hemocyte supernatants both increased to a significantly higher level (2.71- and 2.40-fold, *p* < 0.05) comparing with that in the Neg-Ctrl group at 1 h after LPS stimulation (Figures 1A,B). No significant changes of ACh and NE contents were observed at other time points after LPS stimulation (*p* > 0.05). Our results indicated that LPS stimulation could quickly trigger oyster hemocyte to *de novo* produce cholinergic and adrenergic neurotransmitters.

### Expression and Activity Variations of Key Enzymes in ACh/NE Metabolism after LPS Stimulation

The mRNA expression levels and protein activities of CgChAT, CgDBH, CgAChE, and CgMAO were examined to further ascertain the *de novo* production and degradation of ACh and NE in oyster hemocytes at 30 min and 1 h after LPS stimulation (Figure 1). In general, there were low mRNA expression levels in hemocytes for all the four examined genes under normal status. Except for the activity of CgDBH, the mRNA expressions and protein activities kept relatively stable levels at 30 min in most of the groups, while significant changes were observed at 1 h posttreatment. At 1 h after LPS stimulation, the mRNA expression of CgChAT was significantly upregulated, which was 3.67-fold of that in the Neg-Ctrl group (Figure 1C, *p* < 0.05), and the expression of CgDBH also increased to 4.67-fold of that in the Neg-Ctrl group (Figure 1D, *p* < 0.05). Similarly, the enzyme activities of CgChAT and CgDBH were upregulated to 1.34-fold and 2.04-fold comparing with that in the Neg-Ctrl group at 1 h after LPS exposure (Figures 1E,F, *p* < 0.05). Only in Figure 1F, the activity of CgDBH at 30 min after LPS stimulation was significantly higher (*p* < 0.01) than that in control group.

Conversely, both the mRNA expression levels and protein activities of CgAChE significantly decreased at 1 h after LPS stimulation, which were 0.42-fold and 0.74-fold of that in the Neg-Ctrl group, respectively (Figures 1G,I, *p* < 0.05). As for CgMAO, although its mRNA transcripts in the LPS group remained to a comparable level with that in the Neg-Ctrl group (Figure 1H, *p* > 0.05), its protein activity was severely downregulated to the 0.50-fold of that in the Neg-Ctrl group (Figure 1J, *p* < 0.05). These results revealed the existence of the sophisticated intracellular machinery for the generation, release and inactivation of ACh and NE in oyster hemocytes.

### Phagocytic Activities after Treatments with Receptor Antagonists and LPS

**Figure 2:**
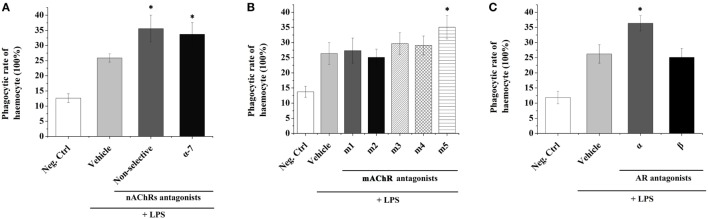
Transmembrane receptors mediate the immune regulation of hemocyte-derived acetylcholine (Ach) and norepinephrine (NE). The immune response (represented by hemocyte phagocytic activity) was induced by lipopolysaccharide (LPS). Adrenoceptors **(A)**, muscarinic **(B)**, and nicotinic **(C)** Ach receptors were blocked pharmacologically to explore their functions in mediating immune regulation of hemocyte-derived ACh and NE. Each bar represents *N* = 6 samples. All data are presented as mean ± SD. Asterisks indicate statistical significance comparing with the vehicle control.

In order to understand the possible mediation of hemocyte-derived ACh to cellular immune response, the phagocytic activity of oyster hemocyte after the incubation of ACh receptor antagonist followed by the LPS stimulation for 3 h was determined (Figure 2). The phagocytic activity of hemocyte in the non-selective nAChR antagonist group (35.6%) was significantly higher than that (25.87%) in Vehicle group in which receptors were not blocked before LPS stimulation (Figure 2A, *p* < 0.05). Significant increase of phagocytic activity was also observed after the block of α-7 nAChR with specific antagonist (33.73 vs. 25.87% in Vehicle group, *p* < 0.05). In addition, for the block of mAChRs, the phagocytic activity increased significantly from 26.4% in Vehicle group to 35.03% in m5 mAChR antagonist group (Figure 2B, *p* < 0.05). No significant changes were observed in m1-4 antagonist treatment groups (Figure 2B, *p* > 0.05).

The phagocytic activities of oyster hemocytes after the incubation of NE receptor antagonist were also determined. The phagocytic activity in Vehicle group (without receptor block before LPS stimulation) was significantly upregulated to 26.23% (Figure 2C, *p* < 0.05) comparing with that in the Neg-Ctrl group (11.87%). After inhibiting AARs, a significant increase of hemocyte phagocytic activity (36.37%) was detected as compared with that in Vehicle group (Figure 2C, *p* < 0.05). However, the phagocytic activity of hemocyte showed no significant change (*p* > 0.05) after the incubation of BAR antagonist. These results suggested that the hemocyte-derived ACh and NE could in turn modulate the immune responses of oyster hemocytes through autocrine/paracrine pathways *via* the mediation of nAChRs, m5 mAChR and AARs.

### Changes of TNF Expressions in Oyster Hemocytes after Receptor Inhibition and LPS Stimulation

**Figure 3:**
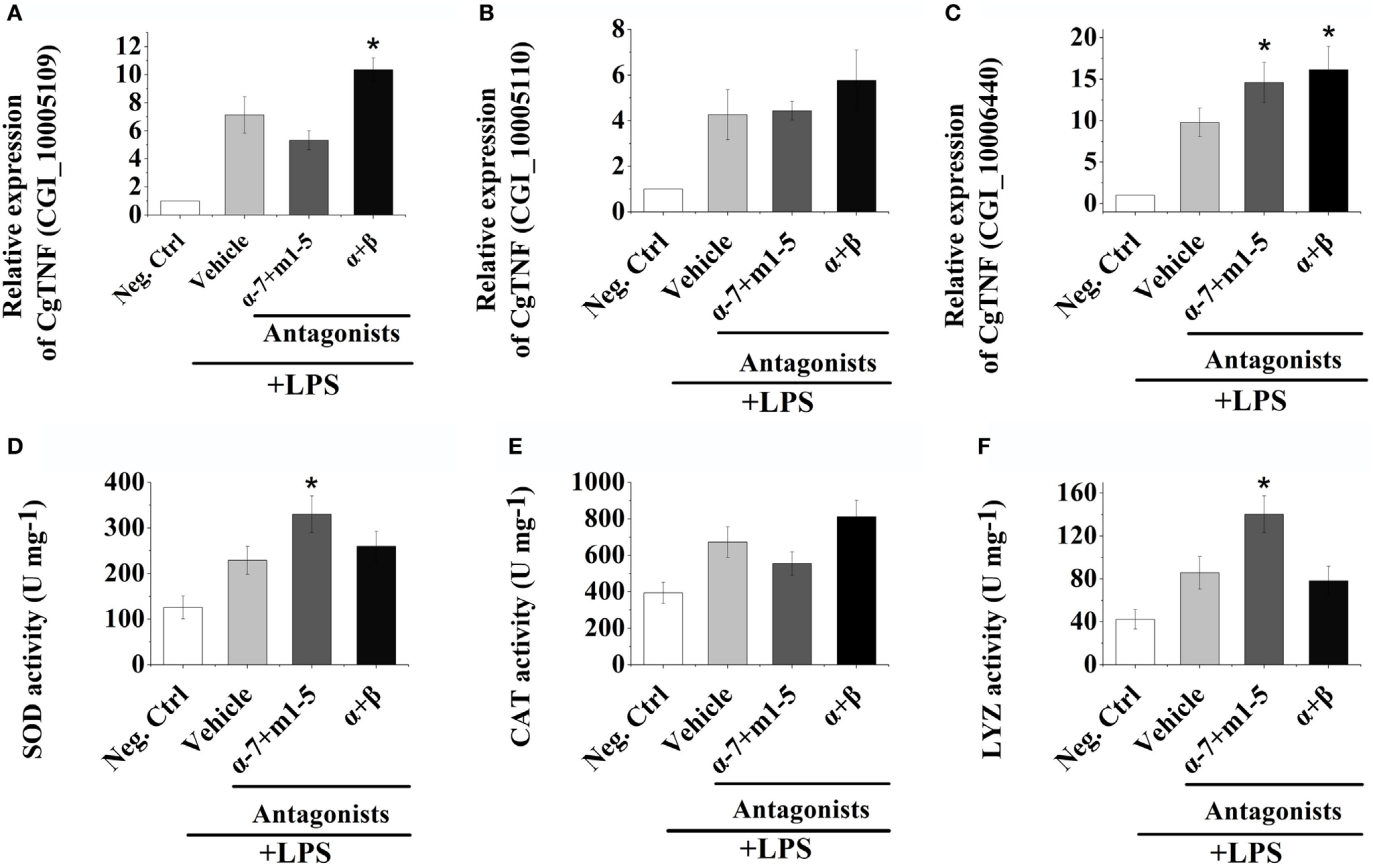
Hemocyte-derived acetylcholine and norepinephrine regulate cellular and humoral immunity. **(A–C)** After stimulation with 100 ng mL^−1^ of lipopolysaccharide (LPS) *in vitro*, mRNA from hemocytes were sampled and subjected to real-time PCR analysates for three oyster tumor necrosis factor genes, CGI_10005109 **(A)**, CGI_10005110 **(B)**, and CGI_10006440 **(C)**. **(D–F)** After stimulation with 100 ng mL^−1^ of LPS *in vitro*, protein from hemocytes were extracted and subjected to enzyme activity measurement of superoxide dismutase **(D)**, catalase **(E)**, and lysosome **(F)**. Each bar represents *N* = 6 samples. All data are presented as mean ± SD. Asterisks indicate statistical significance comparing with the vehicle control.

The mRNA expression levels of three oyster TNFs (CGI_10005109, CGI_10005110, and CGI_10006440) in hemocytes were examined by quantitative real-time PCR after receptor block and LPS stimulations to further explore the autocrine/paracrine immunomodulation patterns in oyster hemocytes. As shown in Figures 3A–C, the mRNA expressions of CGI_10005109, CGI_10005110, and CGI_10006440 all increased to a significant level at 3 h post-LPS stimulation (*p* < 0.05). After the inhibition of ARs on the surface of hemocytes by the incubation of α + β AR antagonists, the mRNA expression of CGI_10005109 and CGI_10006440 was significantly upregulated to 1.45-fold and 1.65-fold that in Vehicle group, respectively (Figures 3A,C, *p* < 0.05). No obvious change of the CGI_10005110 expression level was detected (Figure 3B, *p* > 0.05).

After AChRs were inhibited by nAChRs and mAChRs antagonists, the mRNA expression of CGI_10006440 increased to 1.49-fold of that in Vehicle group (Figure 3C, *p* < 0.05). No obvious variations of the expressions of CGI_10005110 and CGI_10006440 were observed after the incubation of AChRs antagonists (Figures 3A,B, *p* > 0.05). Our results illustrated that hemocyte-derived neurotransmitters could regulate cytokine production in oyster hemocyte through autocrine/paracrine pathways.

### Alteration of Immune-Related Enzyme Activities after Receptor Block and LPS Stimulation

In order to investigate the humoral immune regulation induced by hemocyte-derived ACh and NE, the activities of SOD, CAT, and LYZ were determined postreceptor antagonist incubation and LPS stimulation. As shown in Figures 3D–F, the activities of SOD, CAT, and LYZ were severely upregulated at 3 h post LPS stimulation. After the block of AChRs with antagonists, the activities of SOD and LYZ were significantly increased, which were 1.44- and 1.63-fold of that in Vehicle groups, respectively (Figures 3D,F, *p* < 0.05), while no obvious change was observed in the α + β ARs blocking groups (Figures 3D–F, *p* > 0.05). These results implied that hemocyte-derived neurotransmitters could regulate humoral immunity in oyster through autocrine/paracrine pathways.

## Discussion

Neurotransmitters are traditionally considered as nerve-secreted molecules that trigger or inhibit neurosecretion (40). Yet, it is demonstrated that many vertebrate immune cells can also synthesize and/or release neurotransmitters to regulate immune function by autocrine signaling pathway (2), which offers a novel perspective in revealing the fine-tuning of immune regulation. In the present study, the possible capability of oyster hemocytes to produce cholinergic and adrenergic neurotransmitters was investigated to unveil the possible autocrine mechanism in marine invertebrates, and the immune regulation mediated by these immunocyte-derived neurotransmitters was further explored. ACh and NE concentrations in the cell culture medium significantly increased at 1 h post-LPS stimulation, and these neurotransmitters should be produced by *in vitro* cultured hemocytes. It was reported that noradrenaline and adrenaline levels in supernatants of human macrophages and polymorphonuclear cells increased significantly at 4 h after LPS exposure (6). CD4^+^ T cells contained substantially more ACh compared with CD8^+^ T cells or B cells, while the synthesis and release of ACh from lymphocyte were increased during mitogen (41). The present results indicated that oyster hemocytes can *de novo* produce neurotransmitters such as ACh and NE under LPS stimulation, like those immune cells in vertebrates.

ACh is synthesized from acetyl coenzyme A and choline by the enzyme ChAT (42), while NE is converted from DA by the activity of the enzyme DBH (43). ChAT is known as a rate-limiting enzyme in ACh synthesis (44), whereas DBH is a copper-containing enzyme that uses molecular oxygen and ascorbate to catalyze the addition of a hydroxyl group on the beta-carbon of dopamine to form norepinephrine (45). In the present study, the mRNA expressions and protein activities of CgChAT and CgDBH in hemocytes both increased dramatically at 1 h after LPS stimulation. These results suggested that oyster hemocytes possessed the molecular components to produce ACh and NE post LPS stimulation like mammalian phagocytes (6). Usually, the actions of neurotransmitters are terminated in three ways including reuptake into nerve terminals, diffusion into extracellular fluids and metabolic transformation (6). AChE and MAO are crucial enzymes for the degradation of ACh and NE (2, 22). In the present study, the mRNA expression levels of CgAChE decreased significantly at 1 h after LPS stimulation, and the activities of CgAChE and CgMAO also decreased (Figure 1). These results were consistent with the dramatic increase of ACh and NE concentrations after LPS stimulation (Figures 1A,B), suggesting that the degradation of ACh and NE was inhibited by the abundance of ACh/NE that modulated immune response against LPS stimulation. With the characterization of both synthesizing and degradating enzymes in the metabolism of ACh/NE, our results ascertained the sophisticated cellular machinery for *de novo* generation, release and inactivation of ACh/NE in oyster hemocytes.

Neurotransmitters are critical for the immune modulation by binding to their specific receptors on the surface of immune cells in vertebrates (46). For example, ACh can activate nAChR in macrophages of mammals to inhibit NF-κB signaling, thereby decreasing the production of proinflammatory cytokines, and finally causing severe inflammatory reaction and pathological responses (47). So far, receptors for many neurotransmitters including NE, ACh, ENK, 5-HT, and GABA have also been identified on the surface of bivalve hemocytes (27, 28, 48–50), and this represents the molecular basis for the autocrine immune regulation in mollusks. To investigate the mediation of transmembrane neurotransmitter receptors during autocrine immunomodulation in mollusks, antagonists for ACh and NE receptors were employed to block their binding activities *in vitro*, and the fraction of phagocytizing oyster hemocytes were determined. As shown in our results, the phagocytic activities of oyster hemocytes increased significantly at 1 h after LPS stimulation in non-selective nAChRs antagonist and m5 mAChR antagonist groups. nAChRs and mAChRs are characterized as two subtypes of AChRs based on their affinities and sensitivities to different ligands (nicotine/muscarine) (30, 51). Both nAChRs and mAChRs are critical for the immune modulation such as the production of cytokines and modification of antibody synthesis in humans (52). Low-dose nicotine causes inhibition of TNF-α, prostaglandin E_2_, and macrophage inflammatory protein-1α production in LPS-activated monocytes, and these suppressive effects are mediated through α7nAChR (53). Activation of T cells with phytohemagglutinin (PAH) and phorbol 12-myristate 13-acetate (PMA) upregulates the expression of ChAT and m5 mAChR genes *via* the protein kinase C (PKC) and mitogen-activated protein kinase pathways (54). In addition, it was found that ACh can inhibit phagocyte apoptosis and phagocytosis in oyster (34). Results in the current study suggest that hemocyte-derived ACh can trigger negative autocrine/paracrine immunomodulation in response to LPS stimulation *via* the mediation of nAChR and m5 mAChR. As for the neurotransmitter NE, there were two main groups of receptors, AARs and BARs. AARs include the subtypes α-1 (a Gq-coupled receptor) and α-2 (a Gi-coupled receptor) (55). BARs include the subtypes β-1, β-2, and β-3, and all these three types are linked to Gs proteins (although β-2 also couples to Gi) (56). In the present study, the phagocytic activity of oyster hemocytes was significantly increased after the block of AARs as compared with that in control group, while similar results were not observed when BARs were inhibited. It was reported that AARs were indispensable mediators in the innate immunity. In the rat thymus, A1AR was colocalized with the monocyte/macrophage marker CD68. Functional A1ARs were identified on murine RAW264 macrophages when phenylephrine and other PKC activating agents were used to initiate cell spreading (57). The present results suggested that ACh and NE released from hemocytes could repress the cellular immune response of oyster *via* the mediation of nAChR, m5 mAChR, and AAR.

The production of immune/inflammatory mediators including cytokines, chemokines, reactive oxygen species, and other immune effectors is modulated by activation of neurotransmitter receptors expressed on immune cells (58). For example, ARs can mediate the inhibition of TNF-α production caused by LPS stimulation in human monocytes (59). α7nAChRs have been shown to negatively regulate the synthesis and release of TNF-α in macrophages (60), and stimulation of A2ARs subtype by exogenous or endogenous NE induced the release of TNF-α by murine peritoneal macrophages stimulated with LPS (61). Our previous research found that neurotransmitters in mollusks could also modulate both cellular and humoral immunity, and immune effectors, such as TNF-α, SOD, CAT, and LYZ were produced during the response for neurotransmitter modulation (14, 62). In the present study, the immune regulatory functions of neurotransmitters released from hemocytes were investigated by *in vitro* experiments. LPS stimulation significantly upregulated the mRNA expressions of oyster TNF genes (CGI_10005109, CGI_10005110, and CGI_10006440), and the block of ARs with α + β ARs antagonists caused a significant increase in the mRNA expressions of CGI_10005109 and CGI_10006440, but not CGI_10005110. Inhibiting AChRs with antagonists could obviously increase the mRNA expression level of CGI_10006440. The activities of SOD and LYZ were severely increased after AChRs were blocked with antagonists. Our previous study found that the three TNF genes, especially CGI_10006440, were vital components of the signaling pathway for the neurotransmitter to modulate the endocrine system of oyster (34). Our results demonstrated for the first time that the hemocyte-derived ACh and NE could exert similar cellular and humoral immunomodulation through autocrine/paracrine pathways in oyster. Interestingly, cytokine (such as TNF-α) production was mainly modulated by NE *via* ARs, while the synthesis of SOD and LYZ was basically regulated by ACh through AChRs in oyster hemocytes during autocrine/paracrine immunomodulation. Such results had never been reported in other species and needed further exploration in the future.

**Figure 4:**
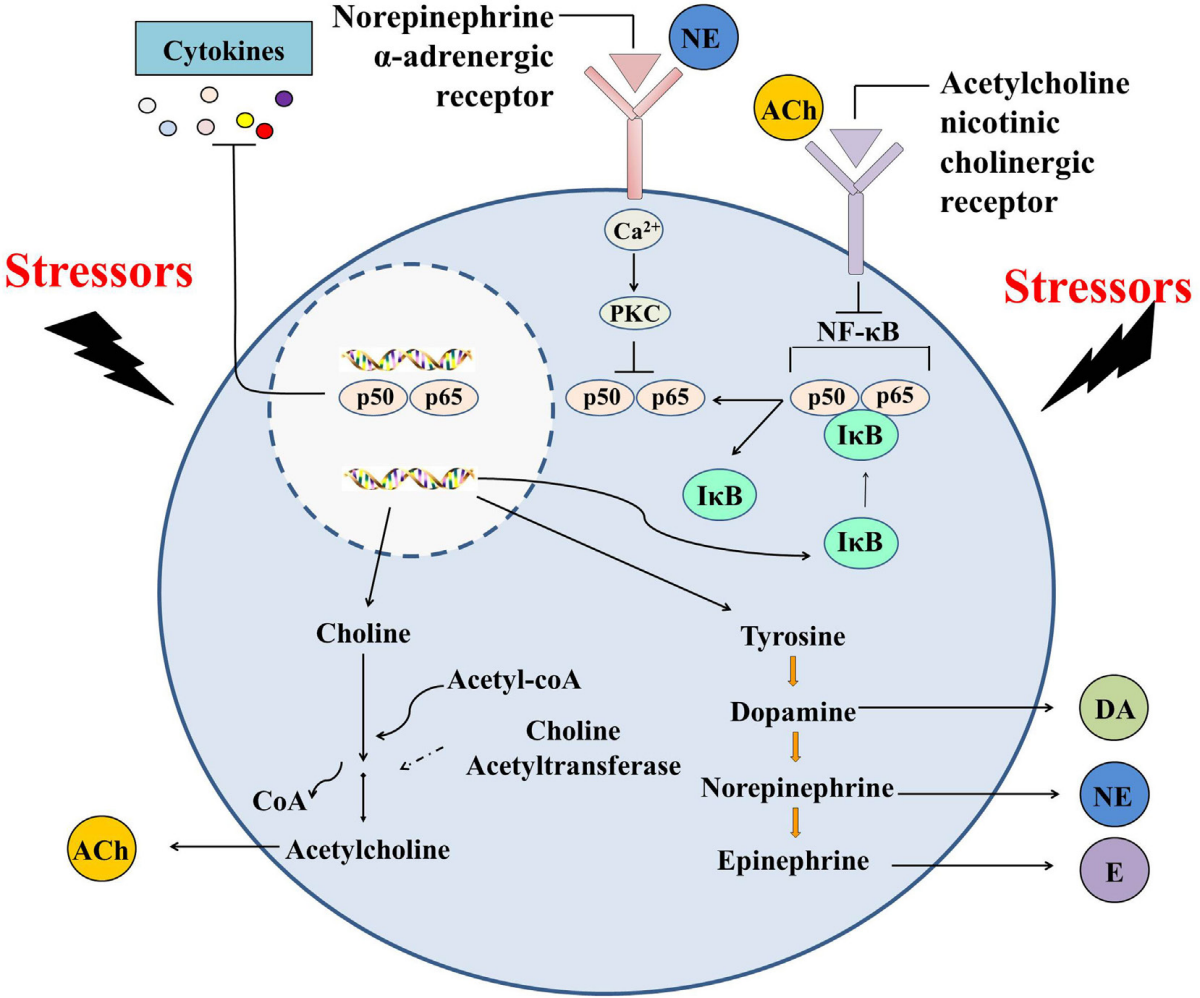
Immunomodulation mediated by hemocyte-derived cholinergic and adrenergic neurotransmitters. Oyster hemocytes should represent similar immune and neuroendocrine functions as their counterparts in vertebrates (e.g., macrophages) and play an indispensable role in autocrine/paracrine immunomodulation, demonstrating that they could serve as suitable model for the study of the origin and evolution of the immune cells.

Taken together, oyster hemocytes are found to be capable of *de novo* synthesizing and releasing ACh and NE, indicating that they are evolutionary primitive immunocytes with the ability to produce cholinergic and adrenergic neurotransmitters. Oyster hemocytes show similar immune and neuroendocrine functions as their counterparts in vertebrates (e.g., macrophages) and play an indispensable role in autocrine/paracrine immunomodulation (Figure 4), demonstrating that they can serve as suitable model for the study of the origin and evolution of the immune cells. With rapid progress in the study of cell typing in mollusks, research should be expanded to explore the variations of neurotransmitter production and the subsequent autocrine/paracrine immunomodulation among different types of oyster hemocytes.

## Ethics Statement

All animal-involving experiments of this study were approved by the Ethics Committee of the Institute of Oceanology, Chinese Academy of Sciences.

## Author Contributions

ZLi, LS, LW, ML, and ZZ conceived and designed the experiments. ZLi performed the experiments. ZLi, ZZ, and QY analyzed the data. LQ contributed reagents/materials/analysis tools. WW and ZLv contributed to the discussion. ZLi, LW, and LS wrote the manuscript. All the authors read and approved the final manuscript.

## Conflict of Interest Statement

The authors declare no competing interests which may be perceived to influence the conduct or analysis of the data.
